# Diagnostic and prognostic utility of SIRI and CAR in necrotizing fasciitis: a retrospective cohort study on disease severity and clinical outcomes

**DOI:** 10.1186/s40001-026-03867-2

**Published:** 2026-01-12

**Authors:** Zheng Xu, Qi Han, Rui Zhang, Yuqin Wang, Guosheng Wang

**Affiliations:** Department of Pancreatic and Biliary Surgery, The First Affiliated Hospital of Harbin Medical University, Harbin, Heilongjiang China

**Keywords:** Disease activity, Necrotizing fasciitis, C-reactive protein/albumin ratio, The systemic inflammation response index, Predictive markers

## Abstract

**Background:**

Necrotizing fasciitis (NF), a fulminant soft tissue infection requiring urgent surgical intervention, presents substantial diagnostic challenges due to its non-specific clinical manifestations during initial stages. This study investigates the prognostic value of two novel inflammatory biomarkers—the systemic inflammatory response index (SIRI) and C-reactive protein-to-albumin ratio (CAR)—in predicting disease progression and clinical outcomes in NF patients.

**Methods:**

This retrospective cohort study analyzed clinical data from 192 patients with confirmed soft tissue infections, comprising 50 necrotizing fasciitis (NF) cases and 142 non-NF presentations. Comparative analysis of SIRI, CAR, and key inflammatory markers was conducted between NF and non-NF cohorts during the perioperative 24-hour period. NF patients underwent severity stratification using Sequential Organ Failure Assessment (SOFA) criteria, categorized as severe (SOFA ≥ 2) or mild (SOFA < 2) subgroups. Univariable and multivariable logistic regression analyses were performed to identify independent risk factors associated with disease severity, and the predictive capacity of these factors was evaluated using receiver operating characteristic (ROC) curve analysis.

**Results:**

The two groups exhibited comparable baseline characteristics and comorbidities, with no statistically significant differences detected (all P > 0.05). Among the 50 NF cases and 142 non-NF cases, the NF group showed significant elevations in inflammatory parameters, coagulation profiles, and metabolic/renal biomarkers compared with non-NF controls. Multivariable regression analysis established CAR, SIRI, blood glucose (GLU), and creatinine (Cr) as independent NF risk factors, showing area under the curve (AUC) values of 0.86, 0.78, 0.68, and 0.65 respectively. In NF severity assessments, CAR and SIRI emerged as independent predictors. ROC curve validation confirmed CAR (AUC = 0.70) and SIRI (AUC = 0.76) as robust severity stratification biomarkers in NF progression assessment.

**Conclusions:**

SIRI and CAR serve as independent risk factors for the development and progression of necrotizing fasciitis, offering novel biomarkers for early diagnosis and prognostic evaluation. These findings could enhance clinical decision-making and optimize patient outcomes.

## Introduction

Necrotizing fasciitis (NF) is a life-threatening invasive infection involving the skin and soft tissues, characterized by rapidly progressive necrosis with a predilection for subcutaneous tissues and fascia. Despite its low prevalence, necrotizing fasciitis carries a mortality rate of 20–30%, with this disparity underscoring the necessity for immediate clinical vigilance in management [1–3]. While epidemiological studies demonstrate geographical disparities in necrotizing fasciitis incidence, this condition consistently associates with predisposing comorbidities including diabetes mellitus, chronic kidney disease, and immunocompromised states. The clinical ambiguity of early-stage NF presentation contributes to delayed therapeutic initiation, consequently elevating mortality rates in affected populations [4, 5]. Therefore, implementing time-sensitive diagnostic protocols and evidence-based therapeutic strategies is critical for optimizing survival outcomes and preserving functional status in NF cohorts.

Contemporary diagnostic frameworks for necrotizing fasciitis necessitate multimodal integration of clinical evaluation, radiographic confirmation, and histopathological verification [6, 7]. Nevertheless, early-stage NF manifestations exhibit limited diagnostic specificity in clinical assessments, while conventional imaging modalities show suboptimal sensitivity during initial diagnostic evaluations [8–10]. Although biopsy offers histopathological confirmation, this diagnostic modality constitutes an invasive intervention with prolonged procedural duration and associated complication risks [11]. Although established diagnostic algorithms including LRINEC (Laboratory Risk Indicator for Necrotizing Fasciitis) and SIARI (Skin Infection and Antibiotic Response Index) demonstrate prognostic value, external validation studies have identified suboptimal sensitivity thresholds and clinically significant false-negative probabilities [12–16]. This imperative necessitates the development of noninvasive diagnostic biomarkers with enhanced sensitivity for early detection and severity stratification in NF, aiming to optimize therapeutic decision-making and prognostic trajectories..

Emerging evidence highlights the growing research significance of the systemic inflammatory response index (SIRI) and C-reactive protein-to-albumin ratio (CAR) as novel inflammatory biomarkers in critical care medicine. The systemic inflammatory response index (SIRI) quantitatively synthesizes granulocyte, lymphocyte, and platelet counts to objectively quantify systemic inflammatory burden, with emerging evidence supporting its utility as a robust prognostic indicator in diverse infectious pathologies [17–19]. The C-reactive protein-to-albumin ratio (CAR) amalgamates acute-phase reactant CRP and nutritional biomarker albumin(ALB), constituting a dual-pathway biomarker that enables multidimensional assessment of inflammatory burden. Accumulating evidence supports the prognostic validity of the C-reactive protein-to-albumin ratio (CAR) in predicting surgical morbidity and long-term survival outcomes across oncological populations [20, 21]. However, the specific roles of SIRI and CAR in NF remain insufficiently explored. This translational study therefore seeks to delineate the prognostic capacity of systemic inflammatory response index (SIRI) and C-reactive protein-to-albumin ratio (CAR) in forecasting NF progression patterns, with the ultimate objective of establishing mechanistic biomarkers for dynamic risk stratification and precision therapeutic interventions.

## Methods

### Patients

This retrospective cohort enrolled 192 patients (50 NF; 142 non-necrotizing controls) from a tertiary care center (January 2022-June 2024). Enrollment employed a standardized EMR-driven screening protocol applying admission diagnostic codes to all hospitalized cases. NF group inclusion required dual surgical-pathological confirmation—surgical identification of necrotic fascia with purulent exudation and pathological verification of neutrophil infiltration, as well as small vessel thrombosis in adipose/fascial tissues—with diagnoses independently reviewed by two senior surgeons; for patients diagnosed with NF, the time frame from hospital admission to surgical intervention was within 12 h. Exclusion criteria comprised incomplete medical records, premature discharge, postoperative follow-up cases, or inter-hospital transfers. Control group inclusion criteria comprised patients with surgically confirmed non-NF soft tissue infections (e.g., abscesses, cellulitis, erysipelas), with exclusion criteria aligned with the NF cohort. Non-NF controls were consecutively enrolled from concurrent soft tissue infection patients. Without pre-matching to NF cases for age, sex, or comorbidities (e.g., diabetes), potential confounders were adjusted for in multivariate models to strengthen inter-group comparison robustness. NF patients were stratified into mild (SOFA < 2) and severe (SOFA ≥ 2) groups using SOFA scores.

### General data

Clinical data from 192 patients with soft tissue infections (41 females, 151 males; age range: 16–91 years, mean age: 54.20 ± 14.38) were analyzed. Baseline data encompassed gender, age, body mass index (BMI), and relevant medical histories, including diabetes, liver disease, kidney disease, cardiovascular diseases, and malignancies. Furthermore, six inflammatory or nutritional indices alongside twelve supplementary hematological parameters were systematically documented. Blood samples were obtained from all patients within 24 h of admission. The calculations for the inflammatory or nutritional indices were as follows:

- C-reactive protein-to-albumin ratio (CAR) = C-reactive protein/Albumin
- Neutrophil-to-lymphocyte ratio (NLR) = Neutrophils/Lymphocytes
- Platelet-to-lymphocyte ratio (PLR) = Platelets/Lymphocytes
- Monocyte-to-lymphocyte ratio (MLR) = Monocytes/Lymphocytes
- Systemic immune-inflammation index (SII) = Platelet count*Neutrophil count/Lymphocyte count
- Systemic inflammatory response index (SIRI) = Monocyte count*Neutrophil count/Lymphocyte count

### Statistical analysis

Statistical analyses and visualization were performed using R (v4.2.3) and GraphPad Prism 8.0. Categorical variables were expressed as frequencies (percentages), while continuous variables were summarized as mean ± standard deviation for normally distributed data or median (interquartile range) for non-normally distributed data. Categorical variables were assessed by chi-square tests. Continuous variables with normal distribution were analyzed via independent-samples t-tests, while non-normally distributed data were evaluated using Mann–Whitney U tests. Univariate and multivariate logistic regression analyses were performed to determine independent risk factors associated with diagnosis and disease stratification. ROC curves were used to assess the predictive performance of each index in NF patients, incorporating sensitivity and specificity evaluations alongside determination of optimal cut-off values for CAR and SIRI using the Youden index (sensitivity + specificity—1). All statistical analyses employed two-tailed tests, with statistical significance defined as P < 0.05.

## Results

### Demographic characteristics and laboratory findings

Demographic and laboratory data for all participants are presented in Table 1, with significant differences (P < 0.05) observed across multiple indices. The NF group demonstrated a significantly higher prevalence of diabetes history (P = 0.028). Laboratory analyses revealed significant differences in inflammatory markers (WBC,PCT, CAR,PLR,NLR, MLR, SII, SIRI,LRINEC scores), coagulation parameters (FIB,D-dimer,PTINR), and metabolic/renal biomarkers (BUN,Cr, GLU).Multivariable regression analysis incorporating significant univariate indices was performed (Table 2). Creatinine (Cr,P = 0.024), Glucose (GLU, P < 0.001), CAR (P < 0.001), and SIRI (P < 0.001) emerged as independent NF risk factors. CAR exhibited the highest odds ratio, underscoring its prognostic significance in NF.

**Table 1:** Demographic, clinical, and laboratory characteristics among different groups

Variables	Non-NF (n = 142)	NF (n = 50)	*P*
Age, Mean ± SD	54.30 ± 14.45	53.92 ± 14.33	0.872
BMI, M (Q₁, Q₃)	25.37 (22.23, 28.64)	25.14 (22.87, 27.44)	0.505
Gender, n(%)			0.501
Female	32 (22.54)	9 (18.00)	
Male	110 (77.46)	41 (82.00)	
Comorbidities, n (%)			
Liver, n(%)			1.000
No	139 (97.89)	49 (98.00)	
Yes	3 (2.11)	1 (2.00)	
Kidney, n(%)			0.838
No	139 (97.89)	48 (96.00)	
Yes	3 (2.11)	2 (4.00)	
Coronary heart disease, n(%)			0.460
No	122 (85.92)	45 (90.00)	
Yes	20 (14.08)	5 (10.00)	
Tumor, n(%)			0.916
No	128 (90.14)	46 (92.00)	
Yes	14 (9.86)	4 (8.00)	
Diabetes, n(%)			**0.028**
No	68 (47.89)	15 (30.00)	
Yes	74 (52.11)	35 (70.00)	
Laboratory tests			
WBC, M (Q₁, Q₃)	12.87 (9.72, 17.62)	22.41 (15.05, 28.73)	** < 0.001**
HGB, M (Q₁, Q₃)	126.50 (110.50, 143.00)	130.00 (110.00, 136.00)	0.741
PLT, M (Q₁, Q₃)	244.50 (173.25, 323.00)	258.00 (170.00, 351.50)	0.821
ALT, M (Q₁, Q₃)	24.15 (17.32, 37.00)	28.10 (19.07, 37.75)	0.323
AST, M (Q₁, Q₃)	24.35 (16.20, 37.21)	24.30 (16.00, 48.95)	0.691
BUN, M (Q₁, Q₃)	6.25 (4.46, 8.67)	9.25 (6.53, 15.23)	** < 0.001**
Cr, M (Q₁, Q₃)	69.37 (54.89, 87.42)	87.25 (63.63, 157.05)	**0.001**
GLU, M (Q₁, Q₃)	7.14 (5.71, 10.39)	14.02 (6.07, 20.12)	** < 0.001**
FIB, M (Q₁, Q₃)	6.56 (5.14, 7.97)	7.75 (6.51, 9.15)	**0.002**
D2, M (Q₁, Q₃)	1.63 (1.00, 2.53)	2.10 (1.34, 3.73)	**0.029**
PTINR, M (Q₁, Q₃)	1.15 (1.07, 1.27)	1.24 (1.12, 1.39)	**0.005**
PCT, M (Q₁, Q₃)	0.81 (0.21, 2.17)	2.75 (1.06, 8.26)	** < 0.001**
CAR, M (Q₁, Q₃)	5.34 (2.47, 8.04)	11.90 (8.08, 14.83)	** < 0.001**
NLR, M (Q₁, Q₃)	9.97 (6.46, 16.25)	21.38 (15.93, 33.10)	** < 0.001**
PLR, M (Q₁, Q₃)	223.03 (151.29, 336.66)	291.34 (217.50, 380.91)	** < 0.001**
MLR, M (Q₁, Q₃)	0.70 (0.44, 0.99)	1.19 (0.82, 1.71)	** <0.001**
SII, M (Q₁, Q₃)	2433.69 (1487.01, 3764.43)	5162.92 (3049.64, 8714.48)	** < 0.001**
SIRI, M (Q₁, Q₃)	7.98 (3.45, 13.35)	21.09 (14.18, 38.17)	** < 0.001**
LRINEC, M (Q₁, Q₃)	5.00 (2.00, 6.00)	7.00 (5.25, 8.00)	** <0.001**

**Table 2:** Multivariate logistic regression results of NF and Non-NF

Variables	β	S.E	Z	*P*	OR (95%CI)
Intercept	− 8.17	1.26	− 6.48	< 0.001	0.00 (0.00 ~ 0.00)
Cr, umol/L	0.01	0.00	2.26	0.024	1.01 (1.01 ~ 1.02)
GLU, mmol/L	0.15	0.04	3.90	< 0.001	1.17 (1.08 ~ 1.26)
CAR ratio	0.35	0.07	5.15	< 0.001	1.41 (1.24 ~ 1.61)
SIRI ratio	0.07	0.02	3.81	< 0.001	1.08 (1.04 ~ 1.12)

*OR* Odds Ratio, *CI* Confidence Interval

### ROC curve analysis of CAR, SIRI, GLU, LRINEC, and Cr for predicting NF

**Fig. 1:**
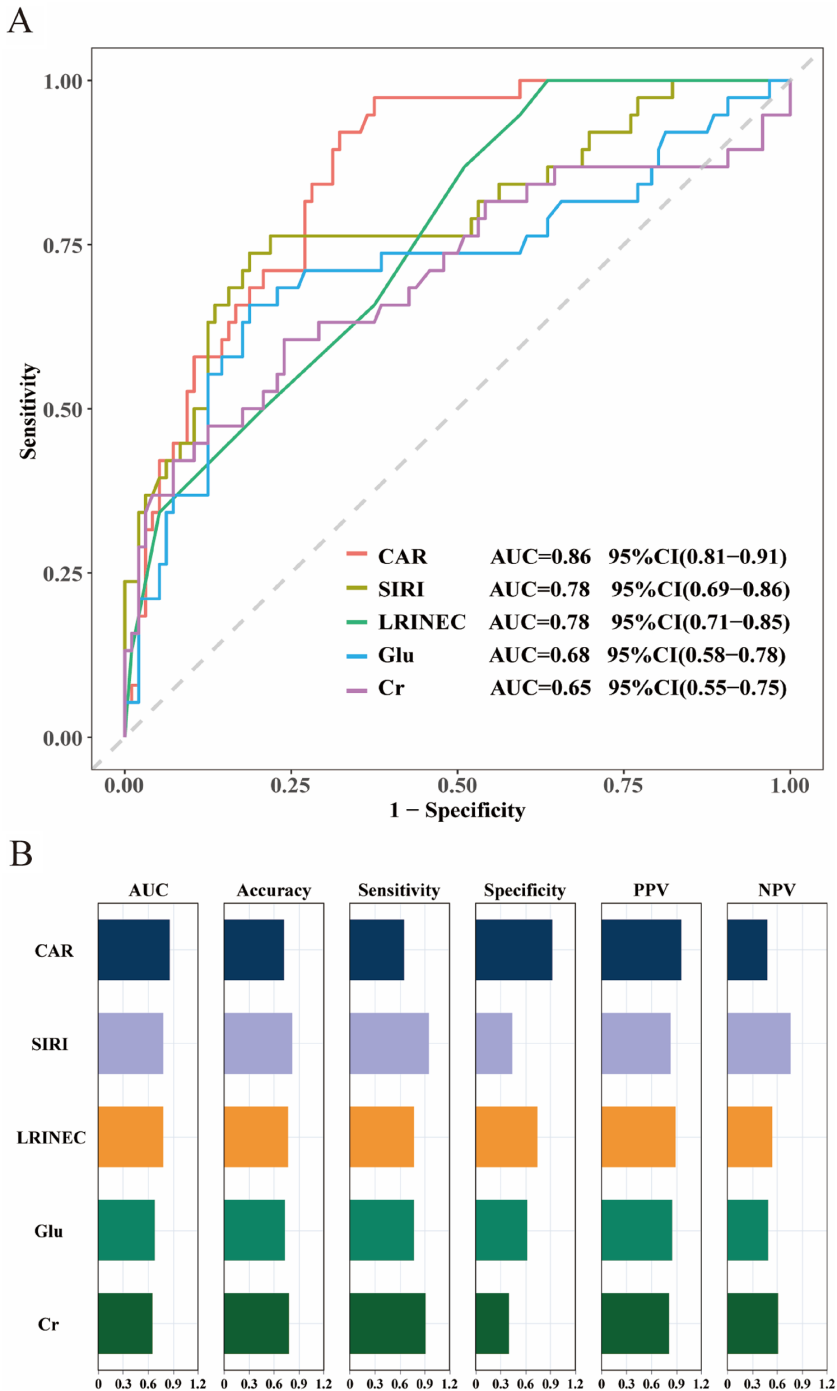
Diagnostic efficacy analysis of different biomarkers for necrotizing fasciitis. **A** ROC curve of the predictive value of relevant indicators for predicting Necrotizing Fasciitis. **B** Visualization of the predictive performance of the five indicators. *CAR* C-reactive protein-to-albumin ratio. *SIRI* Systemic inflammatory response index. *LRINEC* Laboratory Risk Indicator for Necrotizing Fasciitis. *Glu* Glucose. *Cr *Creatinine. *AUC* area under the curve, *PPV* positive predictive Value; and *NPV* negative predictive value

ROC analyses (Fig. 1) assessed the diagnostic performance of multiple indices for NF. CAR demonstrated superior diagnostic accuracy with an AUC of 0.86 (95% CI 0.81–0.91), outperforming SIRI (0.78), LRINEC (0.78), GLU (0.68), and Cr (0.65). Although LRINEC showed superiority in specificity and positive—predictive value (PPV), SIRI exhibited notable advantages in sensitivity, negative—predictive value (NPV), and accuracy. Considering the pivotal significance of early identification of positive cases in clinical practice, SIRI was more advantageous overall and facilitates earlier detection and intervention for the disease.

### Logistic regression analysis of laboratory indices predicting NF severity

Univariate analysis identified significant associations of PCT (P = 0.024), CAR (P = 0.002), and SIRI (P = 0.003) with NF severity (Table 3). Multivariable analysis identified CAR (β = 0.24, P = 0.015; OR = 1.27,95%CI 1.05–1.55) and SIRI (β = 0.03, P = 0.022; OR = 1.03, 95%CI 1.01–1.06) as independent predictors of NF severity. To assess whether diabetes independently influenced disease severity, we incorporated it as a covariate into the multivariate logistic regression model (along with CAR and SIRI). After adjustment, diabetes still showed no independent association with disease severity (OR = 1.03, 95% CI 0.25–4.32, P = 0.968) (Table 4).

**Table 3:** Univariate and multivariate analyses of demographic, clinical, and laboratory characteristics among NF groups with different conditions

Variables	Univariate analyses					Multivariate Analyses				
	β	S.E	Z	*P*	OR (95%CI)	β	S.E	Z	*P*	OR (95%CI)
Age, y	− 0.03	0.02	− 1.62	0.104	0.97 (0.93 ~ 1.01)					
BMI, kg/m^2^	0.10	0.07	1.41	0.158	1.10 (0.96 ~ 1.26)					
Gender										
Famale					1.00 (Reference)					
Male	0.64	0.77	0.83	0.405	1.90 (0.42 ~ 8.67)					
Comorbidities										
Liver										
No					1.00 (Reference)					
Yes	− 15.44	1455.40	− 0.01	0.992	0.00 (0.00 ~ Inf)					
Kidney										
No					1.00 (Reference)					
Yes	− 16.48	1696.73	− 0.01	0.992	0.00 (0.00 ~ Inf)					
Guanxinbing										
No					1.00 (Reference)					
Yes	− 0.27	0.96	− 0.28	0.777	0.76 (0.12 ~ 5.01)					
Tumor										
No					1.00 (Reference)					
Yes	− 1.01	1.19	− 0.85	0.396	0.36 (0.04 ~ 3.76)					
Diabetes										
No					1.00 (Reference)					
Yes	− 0.42	0.62	− 0.68	0.497	0.66 (0.19 ~ 2.21)					
Laboratory tests										
WBC, 10⁹/L	− 0.02	0.03	− 0.48	0.632	0.98 (0.92 ~ 1.05)					
HGB, g/L	− 0.01	0.01	− 0.90	0.370	0.99 (0.96 ~ 1.01)					
PLT, 10⁹/L	− 0.00	0.00	− 0.83	0.407	1.00 (0.99 ~ 1.00)					
ALT, U/L	0.01	0.01	0.74	0.458	1.01 (0.99 ~ 1.02)					
AST, U/L	0.01	0.01	1.59	0.111	1.01 (1.00 ~ 1.03)					
BUN, mmol/L	0.02	0.04	0.55	0.579	1.02 (0.94 ~ 1.11)					
Cr, umol/L	0.00	0.00	0.90	0.369	1.00 (1.00 ~ 1.01)					
GLU, mmol/L	− 0.00	0.03	− 0.04	0.966	1.00 (0.93 ~ 1.07)					
FIB, g/L	− 0.11	0.12	− 0.88	0.381	0.90 (0.71 ~ 1.14)					
D2, mg/L	0.10	0.14	0.75	0.455	1.11 (0.85 ~ 1.44)					
PTINR ratio	1.44	1.26	1.14	0.253	4.21 (0.36 ~ 49.41)					
PCT,ng/mL	0.12	0.05	2.26	**0.024**	1.12 (1.02 ~ 1.24)					
CAR ratio	0.30	0.10	3.13	**0.002**	1.35 (1.12 ~ 1.63)	0.24	0.10	2.43	**0.015**	1.27(1.05 ~ 1.55)
NLR ratio	− 0.01	0.01	− 0.81	0.417	0.99 (0.96 ~ 1.02)					
PLR ratio	− 0.00	0.00	− 0.18	0.858	1.00 (1.00 ~ 1.00)					
SII ratio	− 0.00	0.00	− 0.82	0.414	1.00 (1.00 ~ 1.00)					
SIRI ratio	0.04	0.01	3.00	**0.003**	1.04 (1.01 ~ 1.07)	0.03	0.01	2.29	**0.022**	1.03(1.01 ~ 1.06)
MLR ratio	0.16	0.34	0.46	0.646	1.17 (0.60 ~ 2.29)

**Table 4:** Multivariate logistic regression analysis including diabetes as a covariate

Variables	β	S.E	Z	*P*	OR (95%CI)
Intercept	− 4.37	1.41	− 3.10	**0.002**	0.01 (0.00 ~ 0.20)
CAR ratio	0.24	0.10	2.38	**0.018**	1.27 (1.04 ~ 1.56)
SIRI ratio	0.03	0.01	2.28	**0.023**	1.03 (1.01 ~ 1.06)
Diabetes					
No					1.00 (Reference)
Yes	0.03	0.73	0.04	0.968	1.03 (0.25 ~ 4.32)

*OR* Odds Ratio, *CI* Confidence Interval

### Correlation analysis between inflammatory indices and NF severity

**Fig. 2:**
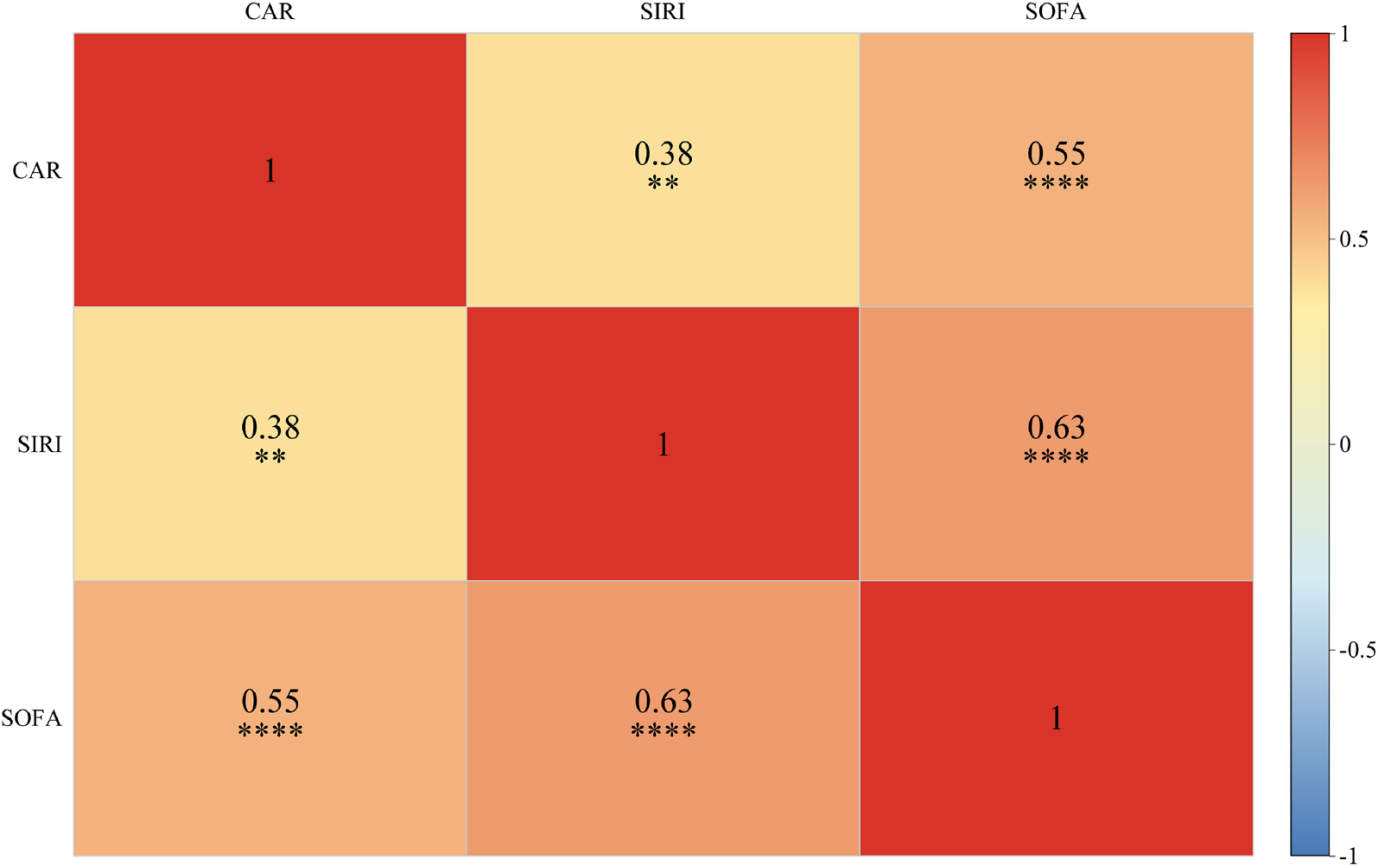
The correlation heatmap among CAR, SIRI, and SOFA

As shown in the Fig. 2, the heatmap demonstrates the correlations among CAR, SIRI, and SOFA. All three exhibit positive correlations pairwise. Among them, the correlation coefficient between CAR and SIRI is 0.38 (P < 0.01), a significant positive correlation; the correlation coefficient between CAR and SOFA is 0.55 (P < 0.0001), and that between SIRI and SOFA is 0.63 (P < 0.0001), with the latter two showing extremely significant positive correlations. This suggests a close association among the three in terms of disease severity or pathological mechanisms, with the strongest correlation between SIRI and SOFA, providing data support for understanding disease mechanisms and clinical evaluation.

### ROC curve analysis of CAR and SIRI for predicting NF severity

**Fig. 3:**
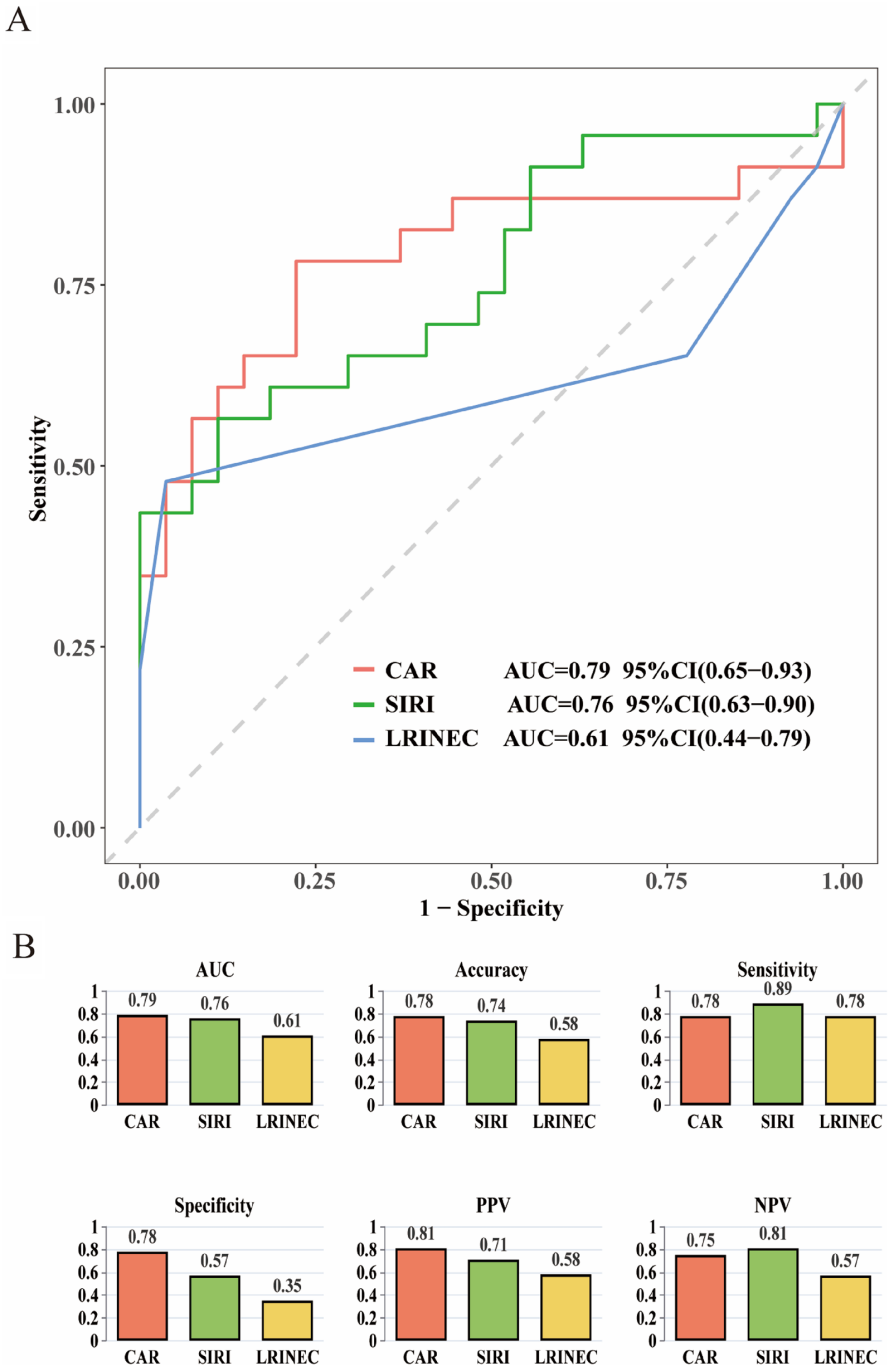
ROC Analysis and Visualization of three NF Severity Predictors. CAR C-reactive protein-to-albumin ratio. *SIRI* Systemic inflammatory response index. *LRINEC* Laboratory Risk Indicator for Necrotizing Fasciitis. *AUC* area under the curve, *PPV* positive predictive Value; and *NPV* negative predictive value

ROC analysis revealed CAR (AUC = 0.79) and SIRI (AUC = 0.76) possessed superior discriminatory capacity for NF severity compared to LRINEC (AUC = 0.61) (Fig. 3).

## Discussion

Necrotizing fasciitis manifests as a fulminant infectious process characterized by fasciocutaneous devastation with progressive tissue necrosis.The paucity of pathognomonic features during initial presentation poses a critical clinical diagnostic challenge, compounding therapeutic delays that substantially elevate mortality risks [22]. This diagnostic need highlights the rationale for developing reliable biomarkers, which is the main goal of this study. Our study demonstrates that SIRI and CAR serve as effective discriminators between NF and non-NF populations, and demonstrate positive correlations with clinical severity gradients, establishing a novel prognostic framework for early clinical detection of high-risk populations.

Suboptimal glycemic control in diabetic patients is established as an independent risk factor for necrotizing fasciitis pathogenesis [2, 4]. Existing evidence demonstrates that 40–71% of NF cases present with documented diabetes history, while hyperglycemia constitutes a key pathogenic determinant predisposing to disseminated infectious complications [23]. Our analysis identified a significantly elevated prevalence of diabetes mellitus in NF patients compared to non-NF counterparts (70.00vs.52.11%; P = 0.028),aligning with established epidemiological observations. Hyperglycemia potentiates bacterial proliferation through enhanced nutrient availability, while sustained glycemic dysregulation induces microangiopathy and peripheral neuropathy, synergistically compromising immunocompetence and impairing tissue repair mechanisms [24]. Despite established links between diabetes and poor outcomes in infectious diseases, our results showed that diabetes was not an independent severity predictor after adjusting for CAR and SIRI. This implies inflammatory biomarkers (CAR, SIRI) may more directly reflect severity—likely due to acute inflammation’s key role in driving necrotizing fasciitis severity, or limitations in sample size/population for detecting diabetes’effects.

Multivariable regression modeling identified CAR (OR = 1.41) and SIRI (OR = 1.08) as independent predictors of NF development, respectively, CAR demonstrated superior discriminative capacity with an AUC of 0.86, significantly outperforming conventional inflammatory markers. This observation corroborates prior investigations documenting CAR's diagnostic superiority in infectious pathology [25–27]. Stratified severity analysis revealed significantly higher CAR (OR = 1.27) and SIRI (OR = 1.03) levels in severe cases (P < 0.01).This implies CAR's potential as a biomarker for NF progression tracking, whereas elevated SIRI signifies progressive immune dysregulation deterioration in critical cases.

The elevated SIRI and CAR in NF may reflect two distinct pathogenic pathways. Specifically, SIRI, which combines monocyte and neutrophil counts, signals uncontrolled systemic inflammation and immune cell dysregulation—a mechanism also observed in other soft tissue infections and sepsis. While direct evidence of SIRI in most other soft tissue infections remains scarce, we refer to sepsis-related studies, which show SIRI is positively correlated with mortality in patients with bloodstream infections (non-linear correlation), and in intensive care unit (ICU) sepsis patients, SIRI correlates with disease severity (assessed via SOFA score) and poor prognosis—with adverse outcomes increasing significantly when SIRI > 6.1 [28, 29]. Of note, its predictive value for sepsis prognosis is better than that of traditional indicators. This can be attributed to SIRI correlating more strongly with SOFA than does CAR—likely because SIRI directly reflects multi-lineage immune cell derangements (neutrophil and monocyte overactivation alongside lymphocyte suppression) that drive organ dysfunction. This dysfunction is not only a key component of SOFA scoring but also, crucially, a core pathological feature of severe necrotizing fasciitis (NF).

Meanwhile, CAR, integrating the acute-phase reactant CRP and albumin as an indicator of nutritional status, implies a catabolic state and weakened host defense. Preclinical research indicates that over—activation of neutrophils and monocytes leads to tissue necrosis through protease and cytokine release, and low albumin levels (hypoalbuminemia) worsen microvascular permeability and facilitate bacterial spread, both of which are critical factors in NF progression. When concurrent elevation of CAR and SIRI beyond thresholds (CAR > 7.17, SIRI > 13.70) is observed in suspected NF patients, urgent surgical intervention (within 6 h) is recommended. For severe NF cases (SOFA ≥ 2), where optimal cut-offs for CAR and SIRI are higher (CAR > 12.01, SIRI > 43.65), delayed surgery (> 12 h) triples mortality, consistent with our data and clinical consensus [30].

Although the LRINEC score demonstrates diagnostic utility, its elevated false-negative rate substantially compromises clinical reliability [31–33]. Our data demonstrate that while the LRINEC score maintains diagnostic value, its clinical applicability is comparatively lower than biomarker-derived indices (SIRI and CAR) in diagnostic accuracy. Clinical investigations have established significant correlations between serum creatinine elevation and necrotizing fasciitis progression, with impaired renal perfusion potentially inducing acute kidney injury that exacerbates systemic deterioration [34]. Notably, creatinine (OR = 1.01) demonstrated limited diagnostic accuracy in the multivariate analysis (AUC = 0.65), possibly reflecting population heterogeneity. In contrast, SIRI and CAR emerged as more reliable biomarkers for early-stage NF detection.

This investigation provides the inaugural evidence supporting the combined diagnostic utility of CAR and SIRI for risk-stratified assessment of necrotizing fasciitis. Clinical protocols should incorporate CAR and SIRI integration into frontline diagnostic algorithms for suspected NF. When concurrent elevation beyond established thresholds is observed, this biochemical profile mandates urgent surgical intervention complemented by empirical antimicrobial coverage. Serial quantification of CAR trajectories enables real-time evaluation of therapeutic response while informing precision adjustments to antimicrobial regimens.

This investigation acknowledges inherent methodological constraints, principally stemming from its monocentric retrospective design, which may compromise external validity due to unmeasured confounding variables and restricted population heterogeneity. The monocentric design and relatively small NF subgroup size (n = 50) not only limit generalizability but also explicitly weaken statistical power—particularly for analyses of the severe NF subgroup (n = 28), further affecting the precision of severity stratification results. The modest cohort size (n = 50 in the NF subgroup) underscores the need for external validation through expanded multicenter cohorts to confirm generalizability. Notably, the NF subgroup (n = 50) may be underpowered for detailed subgroup analyses, particularly in severe NF cases (n = 28), which could affect the precision of severity stratification results. Larger multicenter cohorts are needed to validate these findings. These findings underscore the need for prospective multicentric validation cohorts to facilitate novel biomarker discovery in NF prognostication, thus optimizing clinical translatability across heterogeneous patient demographics.

## Conclusions

In conclusion, CAR and SIRI are effective biomarkers for predicting the occurrence and progression of NF. Specifically, for NF occurrence, CAR has an OR of 1.41 (95% CI 1.24–1.61) and SIRI has an OR of 1.08 (95% CI 1.04–1.12); for NF progression, CAR has an OR of 1.27 (95% CI 1.05–1.55) and SIRI has an OR of 1.03 (95% CI 1.01–1.06). Given that these two biomarkers are positively correlated with NF severity, they can serve as dynamic monitoring tools to facilitate therapeutic stratification. Clinicians should prioritize assessing CAR and SIRI to enable early diagnosis and intervention for NF, thereby improving patient outcomes.

## Data Availability

The data involved in this study can be obtained from the corresponding author.

## Abbreviations

ALT: Alanine aminotransferase
ALB: Albumin
AST: Aspartate aminotransferase
GLU: Blood glucose
BUN: Blood urea nitrogen
BMI: Body Mass Index
CAR: C-reactive protein-to-albumin ratio
Cr: Creatinine
D-dimer: D-Dimer
FIB: Fibrinogen
HGB: Hemoglobin
LRINEC: Laboratory risk indicator for necrotizing fasciitis
MLR: Monocyte-to-lymphocyte ratio
NF: Necrotizing fasciitis
NLR: Neutrophil-to-lymphocyte ratio
PLT: Platelet count
PLR: Platelet-to-lymphocyte ratio
PCT: Procalcitonin
PTINR: Prothrombin time international normalized ratio
ROC: Receiver operating characteristic
SOFA: Sequential organ failure assessment
SIARI: Skin infection and antibiotic response index
SII: Systemic immune-inflammation index
WBC: White blood cell count
